# A novel technique for repairing anterior labral injuries of the shoulder

**DOI:** 10.1308/003588412X13373405387050b

**Published:** 2012-10

**Authors:** JS Weston-Simons, T Singh, DM Ricketts

**Affiliations:** Brighton and Sussex University Hospitals NHS Trust,UK

## BACKGROUND

We describe a novel technique for arthroscopic repair of a Bankart lesion for anterior dislocation of the shoulder, requiring only one anterior arthroscopic portal.

## TECHNIQUE

After anaesthesia, a 30° camera is inserted through a standard posterior shoulder portal. The Bankart lesion is released and mobilised under direct vision through an anterolateral portal using a 6mm cannula. After mobilising the labrum, an anchor is inserted through the anterior portal into the anterior glenoid. A cannulated Tuohy needle (found in most anaesthetic rooms) is used to pass a shuttle suture through the anterior labrum. To mark the correct position a standard 18G needle is first inserted through the anterior capsule anterior to the labrum. Once the desired position has been achieved, the needle is replaced by a longer cannulated Tuohy spinal needle.

**Figure 1:**
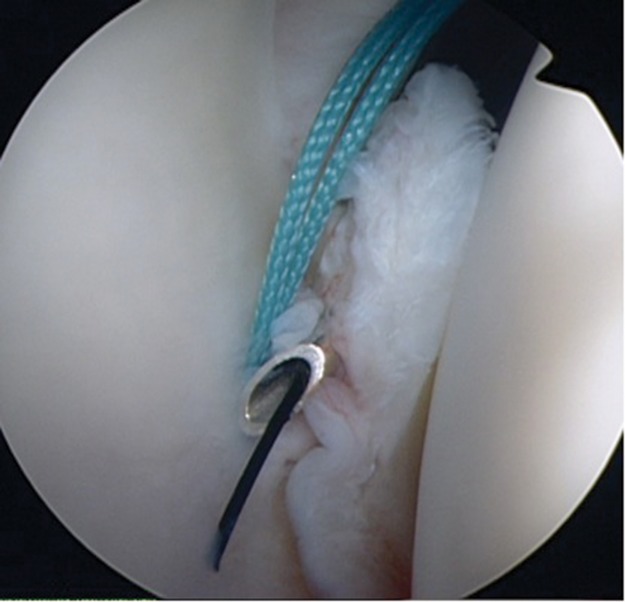
The Tuohy needle and Ethilon® suture have been passed through the labrum. The Mitek® anchor is in place.

Under direct vision, the Tuohy needle is advanced through the capsule and labrum and across the face of the glenoid. If required, graspers can be inserted through the 6mm cannula to aid manipulation of the labrum or the Tuohy needle. A size 0 Ethilon® suture (Ethicon, Somerville, NJ, US) is advanced through the needle and across the face of the glenoid as a shuttle suture (Fig 1), and the labral repair is undertaken in the normal way.

## DISCUSSION

**Figure 2:**
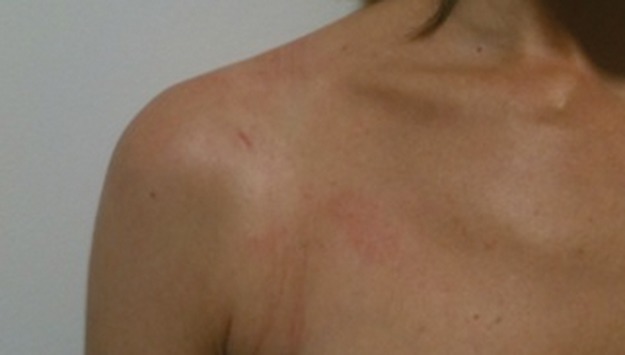
The small anterior scar visible at two weeks

This technique uses readily available equipment and allows access to the inferior labrum as a Tuohy needle is sufficiently slim to pass through the superior part of the subscapularis. Furthermore, it leaves the patient with only one small anterior scar (Fig 2).

